# Clinical outcomes of COVID‐19 treated with remdesivir across the continuum of care

**DOI:** 10.1111/irv.13136

**Published:** 2023-05-17

**Authors:** Christina G. Rivera, Supavit Chesdachai, Evan W. Draper, Richard F. Arndt, Kristin C. Mara, Maria Gonzalez Suarez, Raymund R. Razonable

**Affiliations:** ^1^ Department of Pharmacy Mayo Clinic Rochester Minnesota USA; ^2^ Division of Public Health, Infectious Diseases, and Occupational Medicine Mayo Clinic Rochester Minnesota USA; ^3^ Department of Pharmacy Mayo Clinic Health System Eau Claire Wisconsin USA; ^4^ Department of Quantitative Health Sciences Mayo Clinic Rochester Minnesota USA; ^5^ Division of Nephrology and Hypertension Mayo Clinic Rochester Minnesota USA

**Keywords:** COVID‐19, hospitalization, remdesivir, SARS‐2‐CoV

## Abstract

**Introduction:**

During the early phase of the coronavirus disease 2019 (COVID‐19), remdesivir was only approved for hospitalized patients. Our institution developed hospital‐based, outpatient infusion centers for selected hospitalized patients with COVID‐19 who had clinical improvement to allow for early dismissal. The outcomes of patients who transitioned to complete remdesivir in the outpatient setting were examined.

**Methods:**

Retrospective study of all hospitalized adult patients with COVID‐19 who received at least one dose of remdesivir from November 6, 2020, to November 5, 2021, at one of the Mayo Clinic hospitals.

**Results:**

Among 3029 hospitalized patients who received treatment with remdesivir for COVID‐19, the majority (89.5%) completed the recommended 5‐day course. Among them, 2169 (80%) patients completed treatment during hospitalization, whereas 542 (20.0%) patients were dismissed to complete remdesivir in outpatient infusion centers. Patients who completed the treatment in the outpatient setting had lower odds of death within 28 days (aOR 0.14, 95% CI 0.06–0.32, *p* < 0.001). However, their rate of subsequent hospital encounters within 30 days was higher (aHR 1.88, 95% CI 1.27–2.79, *p* = 0.002). Among patients treated with remdesivir only in the inpatient setting, the adjusted odds of death within 28 days were significantly higher among those who did not complete the 5‐day course of remdesivir (aOR 2.07, 95% CI 1.45–2.95, *p* < 0.001).

**Conclusions:**

This study describes the clinical outcomes of a strategy of transitioning remdesivir therapy from inpatient to outpatient among selected patients. Mortality was lower among patients who completed the 5‐day course of remdesivir.

## 1 INTRODUCTION

Severe acute respiratory syndrome coronavirus 2 (SARS‐CoV‐2), the virus that causes coronavirus disease 2019 (COVID‐19), continues to cause morbidity and mortality globally.^1^ Evidence‐supported treatment for COVID‐19 includes small molecule antiviral drugs, anti‐spike neutralizing monoclonal antibodies, and immunomodulatory agents.^2^ Remdesivir, a direct‐acting nucleotide inhibitor of SARS‐CoV‐2 RNA‐dependent RNA polymerase, was the first antiviral drug approved for clinical use and remains as the backbone for treatment of hospitalized patients with COVID‐19.^3^, ^4^, ^5^

Remdesivir has been demonstrated to be effective for the treatment of hospitalized patients with severe disease and high‐risk outpatients with mild COVID‐19. A phase 3 trial showed that a 5‐day course of remdesivir shortened recovery time among hospitalized patients with COVID‐19.^4^, ^6^, ^7^ Among non‐hospitalized outpatients with conditions that predispose to severe disease progression, a 3‐day course of remdesivir reduced the rates of hospitalization or death by 87% compared to placebo.^8^

Prior to 2022, remdesivir was only approved for clinical use in hospital settings.^9^ At the Mayo Clinic, most patients remained hospitalized for a minimum of 5 days to complete the course of remdesivir treatment. Patients who improved clinically often expressed a desire to leave the hospital prior to completion of the 5‐day course of remdesivir. However, the outcome of the abbreviated course of remdesivir was not known. In November 2021, our program developed hospital‐based infusion therapy centers to allow for earlier dismissal of hospitalized patients who had achieved clinical improvement. Every patient who had clinical improvement, as assessed by the treating hospital providers, was offered an option to continue remdesivir treatment as an outpatient in the hospital‐based infusion facilities. High‐risk patients continued to be monitored using the remote monitoring program. By allowing early dismissal of stable improving hospitalized patients, the healthcare system would benefit by making hospital rooms and staff available for sicker patients.

In this study, we report the outcomes of patients with COVID‐19 treated with remdesivir across the continuum of care. We aim to describe the characteristics and outcomes of patients who were transitioned to complete remdesivir in the hospital‐based outpatient setting and assess their risk of mortality and 28‐day re‐admission rates.

## 2 METHODS

### 2.1 Study design and patient population

This is a retrospective multi‐site cohort study of adult patients during a 1‐year period from November 6, 2020, to November 5, 2021. All patients were admitted to one of the Mayo Clinic hospitals with COVID‐19 diagnosis and received at least one dose of intravenous remdesivir. Per our standard practice, all patients hospitalized for COVID‐19 were assessed for eligibility for a 5‐day remdesivir course in the inpatient setting.

For this study, the patients were categorized into those who: 1) completed the 5‐day remdesivir course entirely inpatient, 2) completed the remdesivir course after transitioning to the outpatient setting, 3) did not complete the remdesivir course but remained inpatient, and 4) did not complete remdesivir after discharge from the hospital. Determination of inpatient or outpatient status was based on patient classes, department, and manual review when unable to determine by other methods. The remdesivir treatment course was categorized as incomplete if <5 doses or <600 mg total was documented in the medication administration record.

Patients were eligible for this study if they were ≥18 years of age and received a first course of remdesivir. If there were >2 calendar days between doses of remdesivir, these were considered separate courses, and the subsequent course(s) were excluded. Other exclusion criteria included receipt of an anti‐spike neutralizing monoclonal antibody or convalescent plasma, use of remdesivir under another study protocol, and patients transitioning from an outside facility during remdesivir treatment. Minnesota residents who declined research authorization were excluded. The Mayo Clinic Institutional Review Board deemed this study to be exempt (IRB 20–012975).

### 2.2 Clinical variables and outcomes

All‐cause mortality was defined as death within 28 days of the first dose of remdesivir. Death related to COVID‐19 was determined by chart review by the study team physician or clinical pharmacist. The second review was performed by the study physician if the cause of death was unable to be determined by the clinical pharmacist.

Liver function tests (LFT, alanine, and aspartate aminotransferases) were reviewed for 10 days from the start of remdesivir treatment. Body mass index (BMI), World Health Organization (WHO) ordinal scale, Monoclonal Antibody Screening Score (MASS), and use of steroids, baricitinib, or tocilizumab were collected. MASS is a clinical risk prioritization score for the allocation of anti‐spike monoclonal antibodies and includes age, BMI, chronic heart, kidney and lung diseases, diabetes mellitus, hypertension, pregnancy, and an immunosuppressed condition or treatment.^10^ Primary diagnosis code was used to identify hospital and emergency care encounters related to COVID‐19 within 30 days of the last administered dose of remdesivir and long COVID‐related encounters within 180 days.

### 2.3 Statistical analysis

Data are summarized using medians and interquartile ranges (IQRs) for continuous data and frequencies and percentages for categorical data. Patient characteristics were compared between groups using Kruskal–Wallis tests for continuous data and either Chi‐square or Fisher's exact tests for categorical data. Multivariable logistic regression was used to assess associations between the treatment group and the outcomes of having an abnormal LFT, all‐cause mortality within 28 days, and death related to COVID‐19 within 28 days of remdesivir treatment initiation after adjusting for age, sex, and MASS. Rates of subsequent ED visits or hospitalization within 30 days and subsequent long COVID encounters within 180 of remdesivir initiation were estimated using the Aalen–Johansen method, where death was considered a competing risk. Cox proportional hazards regression was used to assess the associations between these outcomes and treatment groups. Pairwise comparisons between groups were adjusted for multiple comparisons using the false discovery rate method. All analyses were performed using SAS version 9.4 software (SAS Institute, Inc; Cary, NC).

## 3 RESULTS

### 3.1 Clinical characteristics of the study population

The study population consisted of 3029 patients who were hospitalized and received at least one dose of remdesivir for COVID‐19 during the 1‐year study period. The median age was 67 years [IQR 55, 77]; 1293 (42.7%) were female and 2684 (88.6%) were White. Median BMI was 31 [IQR 27, 37] kg/m^2^. The median MASS was 3 [IQR 2, 6]. The most common medical comorbidities were hypertension (38.1%), cardiovascular disease (33.6%), and diabetes mellitus (31.7%).

Systemic steroids and other immunomodulators (baricitinib or tocilizumab) were used in 2384 (78.7%) and 381 (12.6%) patients, respectively. Most patients (84.1%) required oxygen supplementation, as follows: 1373 (45.3%) by nasal cannula, 829 (27.4%) by high‐flow nasal cannula or non‐invasive positive‐pressor ventilator support, 152 (5.0%) by a mechanical ventilator, and 5 (0.2%) by extracorporeal membrane oxygenation (ECMO). The details of the demographic and other clinical characteristics of patients are in Table 1.

**TABLE 1:** Patient demographics and other clinical characteristics of patients with COVID‐19 categorized by the remdesivir completion status.

	Total (*N* = 3029)	Inpatient/complete (*N* = 2169)	Inpatient/incomplete (*N* = 296)	Outpatient/complete (*N* = 542)	Outpatient/incomplete (*N* = 22)	Overall *p* value	Inpatient/complete versus inpatient/incomplete	Inpatient/complete versus outpatient/complete	Inpatient/incomplete versus outpatient/incomplete	Outpatient/complete versus outpatient/incomplete
Age (years), Median (IQR)	67 (55, 77)	69 (57, 79)	67 (53, 79)	61 (48, 72)	57 (46, 67)	<0.001	0.075	<0.001	0.038	0.36
Sex						0.061	0.53	0.056	0.33	0.24
Female	1293 (42.7%)	951 (43.8%)	124 (41.9%)	206 (38.0%)	12 (54.5%)					
Male	1736 (57.3%)	1218 (56.2%)	172 (58.1%)	336 (62.0%)	10 (45.5%)					
Race/ethnicity						0.33	0.24	0.34	0.89	0.89
White	2684 (88.6%)	1931 (89.0%)	253 (85.5%)	478 (88.2%)	22 (100.0%)					
American Indian/Alaskan Native	13 (0.4%)	10 (0.5%)	0 (0.0%)	3 (0.6%)	0 (0.0%)					
Asian	59 (1.9%)	35 (1.6%)	9 (3.0%)	15 (2.8%)	0 (0.0%)					
Black or African American	70 (2.3%)	48 (2.2%)	12 (4.1%)	10 (1.8%)	0 (0.0%)					
Hispanic/Latino	141 (4.7%)	100 (4.6%)	16 (5.4%)	25 (4.6%)	0 (0.0%)					
Native Hawaii/Pacific Islander	4 (0.1%)	1 (0.0%)	1 (0.3%)	2 (0.4%)	0 (0.0%)					
Other	20 (0.7%)	12 (0.6%)	3 (1.0%)	5 (0.9%)	0 (0.0%)					
Unknown	38 (1.3%)	32 (1.5%)	2 (0.7%)	4 (0.7%)	0 (0.0%)					
BMI						<0.001	0.006	<0.001	0.013	0.42
Missing	155	115	19	18	3					
Median (IQR)	31 (27, 37)	31 (26, 37)	30 (26, 35)	33 (28, 38)	34 (30, 37)					
Baricitinib or tocilizumab	381 (12.6%)	359 (16.6%)	16 (5.4%)	6 (1.1%)	0 (0.0%)	<0.001	<0.001	<0.001	0.35	0.62
Steroid	2384 (78.7%)	1809 (83.4%)	195 (65.9%)	364 (67.2%)	16 (72.7%)	<0.001	<0.001	<0.001	0.59	0.59
MASS						<0.001	0.074	<0.001	0.36	0.55
Missing	8	5	3	0	0					
Median (IQR)	3 (2, 6)	4 (2, 6)	3 (1, 6)	3 (1, 5)	2 (1, 5)					
MASS components:										
Cardiovascular disease	1019 (33.6%)	764 (35.2%)	90 (30.4%)	156 (28.8%)	9 (40.9%)	0.018	0.20	0.020	0.30	0.29
Diabetes mellitus	960 (31.7%)	739 (34.1%)	88 (29.7%)	130 (24.0%)	3 (13.6%)	<0.001	0.19	<0.001	0.19	0.26
Respiratory	678 (22.4%)	520 (24.0%)	58 (19.6%)	97 (17.9%)	3 (13.6%)	0.008	0.19	0.012	0.61	0.61
Renal	151 (5.0%)	124 (5.7%)	15 (5.1%)	12 (2.2%)	0 (0.0%)	0.006	0.65	<0.001	0.56	0.64
Age 65 years and older	1698 (56.1%)	1310 (60.4%)	160 (54.1%)	220 (40.6%)	8 (36.4%)	<0.001	0.074	<0.001	0.15	0.69
Hypertension	1154 (38.1%)	853 (39.3%)	111 (37.5%)	183 (33.8%)	7 (31.8%)	0.11	0.79	0.068	0.79	0.85
BMI	1010 (33.3%)	685 (31.6%)	70 (23.6%)	242 (44.6%)	13 (59.1%)	<0.001	0.008	<0.001	<0.001	0.18
Immunocompromised	413 (13.6%)	304 (14.0%)	42 (14.2%)	66 (12.2%)	1 (4.5%)	0.41	0.94	0.37	0.37	0.37
Pregnant	4 (0.1%)	2 (0.1%)	1 (0.3%)	1 (0.2%)	0 (0.0%)	0.72	0.84	0.84	0.84	0.84
WHO oxygenation scale						<0.001	<0.001	<0.001	0.41	0.41
3—Hospitalized, no oxygen	464 (15.3%)	232 (10.7%)	71 (24.0%)	158 (29.2%)	3 (13.6%)					
4—Mask nasal canula	1373 (45.3%)	924 (42.6%)	122 (41.2%)	314 (57.9%)	13 (59.1%)					
5—High flow noninvasive	829 (27.4%)	706 (32.5%)	55 (18.6%)	66 (12.2%)	2 (9.1%)					
6—Invasive	152 (5.0%)	143 (6.6%)	8 (2.7%)	1 (0.2%)	0 (0.0%)					
7—ECMO	5 (0.2%)	5 (0.2%)	0 (0.0%)	0 (0.0%)	0 (0.0%)					
8—Expired	187 (6.2%)	157 (7.2%)	30 (10.1%)	0 (0.0%)	0 (0.0%)					
Missing	19 (0.6%)	2 (0.1%)	10 (3.4%)	3 (0.6%)	4 (18.2%)					

Abbreviations: BMI, body mass index; ECMO, extracorporeal membrane oxygenation; IQR, interquartile range, MASS, Monoclonal Antibody Screening Score; WHO, World Health Organization.

### 3.2 Overall outcomes of the study population

**FIGURE 1:**
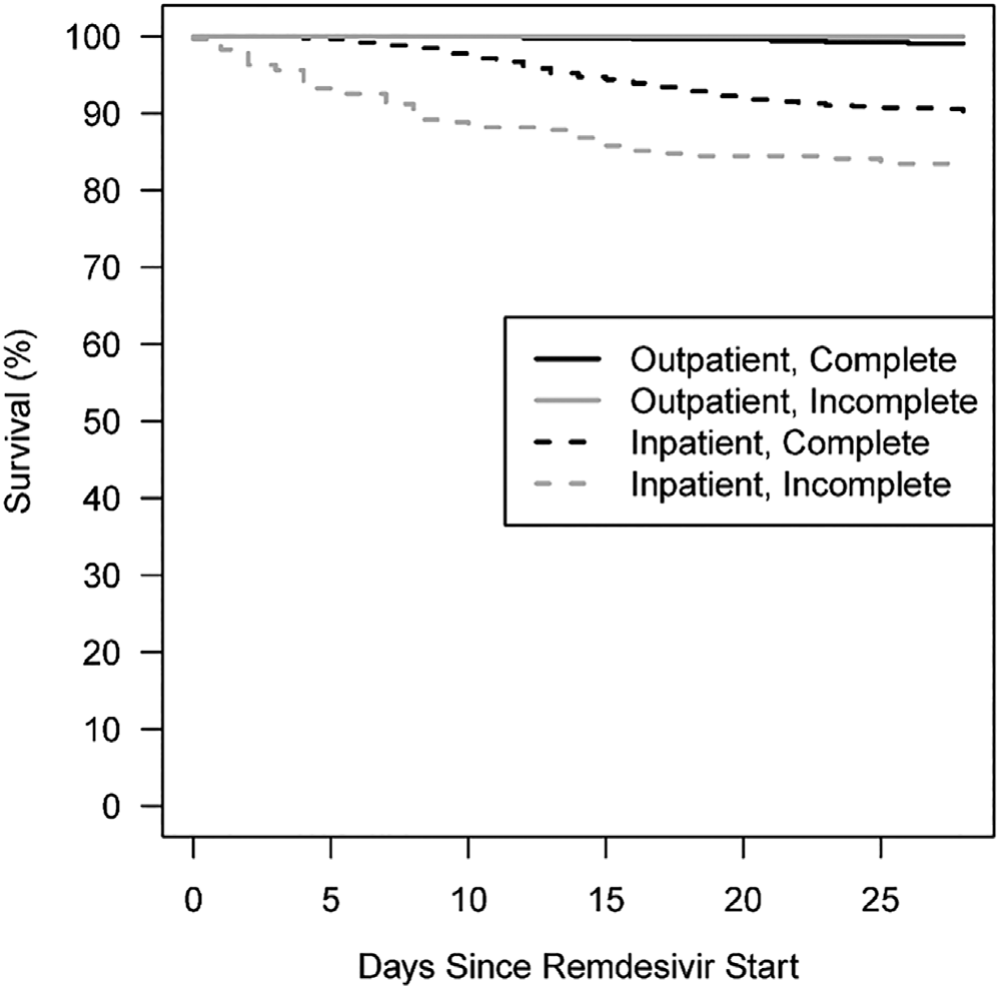
Kaplan–Meier curves depicting survival by remdesivir completion status.

Two hundred and sixty‐five (8.7%) patients died within 28 days of starting remdesivir; 219 (82.6%) were COVID‐19 related. Older age was significantly associated with higher rates of all‐cause and COVID‐19‐related deaths (Table 2). Higher all‐cause and COVID‐19‐related death was also observed among patients who did not complete the 5‐day course of remdesivir (Figure 1). Patients who improved clinically and were dismissed to complete the remdesivir course in the outpatient setting had a lower risk of all‐cause and COVID‐19‐related death (Table 2).

**TABLE 2:** Associations between patient variables and clinical outcomes.

	Abnormal LFTs		Subsequent ED/hospital encounter		Death within 28 days (all‐cause)		COVID‐9‐related death within 28 days	
	Odds ratio^a^ (95% CI)	*p* value	Hazard ratio^b^ (95% CI)	*p* value	Odds ratio^a^ (95% CI)	*p* value	Odds ratio^a^ (95% CI)	*p* value
**Group**								
Inpatient/complete	Reference		Reference		Reference		Reference	
Inpatient/incomplete	0.56 (0.43–0.72)	<0.001	1.58 (0.93–2.70)	0.092	2.07 (1.45–2.95)	<0.001	1.86 (1.27–2.72)	0.001
Outpatient/complete	0.39 (0.32–0.47)	<0.001	1.88 (1.27–2.79)	0.002	0.14 (0.06–0.32)	<0.001	0.04 (0.01–0.22)	<0.001
Outpatient/incomplete	0.30 (0.12–0.72)	0.008	0.65 (0.04–10.69)	0.76	0.39 (0.02–7.03)	0.52	0.44 (0.02–8.09)	0.58
**Age (per 10 years)**	0.89 (0.84–0.93)	<0.001	1.08 (0.96–1.22)	0.22	1.77 (1.59–1.97)	<0.001	1.69 (1.51–1.89)	<0.001
**Sex**								
Female	Reference		Reference		Reference		Reference	
Male	1.46 (1.26–1.70)	<0.001	1.28 (0.91–1.81)	0.16	1.26 (0.96–1.64)	0.096	1.31 (0.98–1.75)	0.066
**MASS (per 1 point)**	0.92 (0.89–0.94)	<0.001	1.06 (1.00–1.12)	0.059	0.97 (0.92–1.01)	0.15	0.99 (0.94–1.04)	0.60
**c‐statistic**	0.65		0.60		0.76		0.76	

Abbreviations: CI, confidence interval; COVID, coronavirus disease; ED, emergency department; LFT, liver function test; MASS, Monoclonal Antibody Screening Score.

^a^ Multivariable logistic regression.

^b^ Multivariable Cox proportional hazards regression.

Among patients who survived during the COVID‐19 hospitalization, 141 (5.1%) patients had subsequent ED visits or re‐admission within 30 days. There was a higher rate of ED visits and re‐admissions within 30 days among patients who were dismissed prior to the completion of 5 days of remdesivir (Table 2). Twenty‐two (0.8%) patients had healthcare encounters due to long COVID within 180 days.

### 3.3 Characteristics and outcomes of patients who completed remdesivir inpatient versus those who were transitioned to the outpatient setting

Of 3029 patients, the majority (2711; 89.5%) completed the remdesivir for a total of 5 days. Among 2711 patients, the majority (*n* = 2169 [80%]) completed the 5 days of remdesivir during hospitalization, whereas 542 patients (20.0%) were dismissed early and transitioned care to complete remdesivir in the outpatient setting. There was a total of 1077 doses of remdesivir infused in hospital‐based outpatient infusion centers.

Patients who transitioned to complete the remdesivir course as an outpatient were significantly younger than those who remained hospitalized (median age, 61 [IQR 48, 72] vs. 69 [IQR 57, 79]; *p* < 0.001), and they had a significantly lower number of comorbidities, as shown by MASS (median MASS 3 [IQR 1, 5] vs. 4 [IQR 2, 6]; *p* < 0.001). The proportion of patients who required oxygen supplementation and systemic corticosteroids and immunomodulators was significantly higher among patients who remained in the inpatient setting (*p* < 0.001). Abnormal liver tests were observed more often among patients who remained in the hospital (*n* = 1315 [60%]) compared to those who transitioned to the outpatient setting (*n* = 231 [42.6%]).

The 28‐day mortality rate was 9.7% (*n* = 211 patients) and 0.9% (*n* = 5 patients) for the inpatient and outpatient groups, respectively. Adjusting for age, sex, and MASS, the outpatient groups had lower odds of death within 28 days (adjusted odd ratio [aOR] 0.14, 95% confidence interval [CI] 0.06–0.32, *p* < 0.001). However, the rate of subsequent ED visits or re‐admission within 30 days was significantly higher in patients dismissed early to complete the remdesivir course in the outpatient setting (adjusted hazard ratio [aHR]: 1.88, 95% CI 1.27–2.79, *p* = 0.002) (Table 2). After excluding those who died in the hospital, there was no significant difference in the odds of death within 28 days between the two groups (aOR: 0.69, 95% CI 0.28–1.68, *p* = 0.41), but the rate of ED visits or re‐admission within 30 days remained higher in the outpatient cohort (aHR: 1.75, 95% CI 1.15–2.64, *p* = 0.009) (Table S1).

### 3.4 Characteristics and outcomes of patients who completed versus did not complete remdesivir during hospitalization

Of 3029 patients, the majority (*n* = 2465 [81.4%]) received remdesivir only in the hospital setting. Among these 2465 patients, 296 (12.0%) did not complete the recommended 5‐day course of remdesivir in the hospital (or in the outpatient setting). Twenty‐two of 296 (7.4%) patients did not complete remdesivir because they died within 5 days after starting treatment.

There were no statistically significant differences in age, sex, and MASS between the inpatients who did and did not complete remdesivir in the hospital. However, the proportion of patients who required oxygen supplementation and received steroids or immunomodulators was significantly higher among patients who completed the remdesivir course for 5 days (*p* < 0.001).

The adjusted odds of the abnormal liver panel were lower among hospitalized patients who did not complete versus those who completed the 5‐day course of remdesivir (aOR 0.56; 95% CI 0.43–0.72, *p* < 0.001). There was no significant difference in subsequent ED visits or re‐admission within 30 days, but the adjusted odds of 28‐day mortality were two‐fold higher among patients who did not complete remdesivir in the hospital (aOR 2.07, 95% CI 1.45–2.95, *p* < 0.001) (Table 2). The results were similar, after excluding the patients who died in the hospital (Table S1).

### 3.5 Characteristics and outcomes of dismissed patients who completed versus did not complete remdesivir in the outpatient setting

A total of 564 patients were dismissed from the hospital prior to the completion of the 5‐day course of remdesivir. Among them, 22 (0.7%) patients received at least one dose of outpatient remdesivir but did not complete the full 5‐day course. There were no statistically significant differences in the clinical characteristics between patients who completed versus those who did not complete outpatient remdesivir.

The all‐cause and COVID‐19 mortality among 22 patients who did not complete the remdesivir course in the outpatient setting was similar to those who remained hospitalized for the 5‐day course of remdesivir. In contrast, the all‐cause and COVID‐19 mortality among those who completed the 5‐day course of remdesivir in the outpatient was lower than those who remained in the hospital and those who were dismissed from the hospital but did not complete the course in the outpatient setting (Table 2).

## 4 DISCUSSION

This retrospective study of a large cohort of hospitalized patients with COVID‐19 treated with remdesivir in a large healthcare system highlights several noteworthy observations. First, one in five hospitalized patients with COVID‐19 improved clinically prior to completion of the 5‐day course of remdesivir treatment. Second, there was a higher rate of hospital re‐admission or ED visits among those who were dismissed prior to completion of the standard 5‐day course of remdesivir. Third, mortality at day 28 was associated with older age and having not completed the 5‐day course of remdesivir. As the COVID‐19 pandemic established endemicity worldwide, our clinical observations have potential applications in its management across the continuum of care settings.

Our study examined all hospitalized patients with COVID‐19 treated with remdesivir from November 2020 to November 2021, a period that encompasses the pre‐vaccination period to the sequential rollout of vaccines to eligible patients.^11^, ^12^ Among our cohort of >3000 patients, the mortality rate was 8.7%; this reflects the high‐risk characteristics of our population. Our patients are predominantly older (56% are older than 65 years old) and had multiple medical comorbidities (as measured by MASS). Hypertension, cardiovascular disease, and diabetes were the most common underlying conditions. Although medical comorbidities have been associated with severe outcomes from COVID‐19, an older age was the most significant risk factor for all‐cause and COVID‐19‐related death in this study.^13^ The significant association between older age and severe outcome from COVID‐19 is well described.^14^, ^15^, ^16^ Since the conduct of our study, there have been increasing rates of vaccination among the population, whereas treatment options have expanded to reduce the risk of death and severe disease from COVID‐19.

In our cohort, one in five patients who required hospitalization for COVID‐19 improved early prior to completion of the standard 5‐day remdesivir treatment. This is a novel observation that has important clinical, operational, and logistical implications. During the different waves of the COVID‐19 pandemic, especially during the SARS‐CoV‐2 Delta wave, healthcare systems were overburdened with a high number of patients requiring medical care in the hospital. This resulted in a shortage of medical beds and staff to care for the sick hospitalized patients. In this situation, one wonders if patients who have improved but have not yet completed the remdesivir course could be safely dismissed from the hospital. Because of the uncertainty of their outcomes, our program established hospital‐based outpatient infusion centers to allow for hospital dismissal of stable and improving patients yet provided the infrastructure to complete the treatment course. This strategy is akin to well‐established outpatient antimicrobial therapy (OPAT) programs, where intravenous antibiotics are administered in infusion units outside the hospital settings. As a result of this practice, we estimated a total of 1077 hospital rooms that were made available to other patients needing critical services in the hospital.

This study observed that the rate of death by day 28 among patients who did not complete the 5‐day course of remdesivir was higher compared to those who completed the treatment course. In particular, patients who did not complete the remdesivir course in the hospital and did not receive an additional dose of remdesivir as an outpatient had three‐time higher odds of death within 28 days compared to patients who completed the treatment course. It is possible that this outcome could be accounted for by 22 patients who died during the treatment with remdesivir; however, these patients constitute only the minority of those who did not complete the treatment. Moreover, it also appears that the survival rate of patients who completed the course in the outpatient setting was better than those who were dismissed from the hospital but did not complete the full 5‐day remdesivir course. Our findings therefore imply that completion of a planned 5‐day course of remdesivir, whether it is in the hospital or in the outpatient setting, is crucial to improving the outcomes. Physical and electronic infrastructures to ensure the administration of intravenous remdesivir in the outpatient setting are therefore important. The implementation of our strategy was intertwined with the establishment of the Monoclonal Antibody Treatment (MATRx) Program in November 2020.^17^ Since the infrastructure of outpatient remdesivir infusion was operational since November 2020, it was an easy transition for our program to provide remdesivir as an approved option for outpatients with mild to moderate COVID‐19. Our program has used the 3‐day outpatient remdesivir course in patients with mild to moderate COVID‐19 within 7 days of symptom onset, especially among patients with contraindications to the use of oral ritonavir‐boosted nirmatrelvir and during the period when there was the scarcity of effective anti‐spike neutralizing monoclonal antibodies (at a time when it was still authorized for use).^10^ In this context, it is important to emphasize the need for patients to be diagnosed early during the mild phase, when the disease pathogenesis is driven by viral replication, so that oral or intravenous antiviral drug therapies are provided early to halt clinical disease progression. Among patients who needed hospitalization for severe disease, however, our report describes the option for continued treatment in the outpatient setting for those who improve clinically prior to 5 days of remdesivir treatment. The structure and the lessons learned during the implementation of our outpatient remdesivir program have proven crucial in our institutional efforts at pandemic preparedness and may also serve as a lesson for other institutions.

Despite the benefits of early dismissal (by allowing patients to recover in the comfort of their home environment), it is important to point out that there was a higher rate of re‐admissions or ED visits among patients who were dismissed early to complete remdesivir in the outpatient setting. We have mitigated this risk by enrolling our high‐risk patients in a remote monitoring program, where patients are dismissed with gadgets that will measure their oxygenation status and other vital signs. Although it is possible that clinically improving patients have been prematurely dismissed, the biphasic course of COVID‐19 could have prompted patients to seek further care when their symptoms rebounded.^18^ This observation further calls into question the assumptions that patients with COVID‐19 selected for hospital dismissal are well enough that ongoing medical care, including medication therapy, is unneeded. It is therefore important to remotely monitor these patients at home, in order to capture early warning signs that would require further medical intervention.^19^ It will also be important to further characterize these patients and define risk factors for re‐admissions in order to mitigate the need for further medical evaluations.

There are several limitations of this study. First, it is a retrospective study with inherent deficiencies in capturing all clinically relevant information for all patients. We did not collect the specific reasons for not completing the 5‐day treatment course; some of these reasons may have accounted for our study findings. Second, the cohort was stratified patients into four groups, with varying demographic and clinical characteristics. We attempted to adjust for them in the multivariable analysis. Third, the findings may only apply to healthcare systems with the infrastructure that allows for the safe administration of remdesivir in the outpatient setting. Although our program adapted this novel strategy in conjunction with the implementation of anti‐spike monoclonal antibody infusion, the logistics of scheduling once daily doses of remdesivir for patients was challenging (but not impossible) to implement. It will be important to assess the economic impact of this practice as well as patient perception and satisfaction with this strategy of care. Fourth, selection bias and other unmeasured confounders are possible in this retrospective study. For example, the reason for not completing the course was not able to be considered in the outcome analysis.

This study of over 3000 patients hospitalized for COVID‐19 highlights the lower mortality rates among patients who completed a 5‐day course of remdesivir treatment when compared to those who did not. Older age was significantly associated with a higher risk of death, whereas completion of the full course of remdesivir was associated with a lower risk of death. Among patients who have improved clinically to allow for early hospital dismissal, it is important to ensure that they complete their treatment course in the outpatient setting. This novel strategy of transitioning remdesivir treatment among hospitalized patients from inpatient to outpatient settings appears to be associated with clinical benefits, in addition to decongesting an overburdened hospital system. This strategy could be implemented selectively among stable patients, especially when hospitals are overburdened with a census exceeding their capacity for care.

## AUTHOR CONTRIBUTIONS

**Christina G. Rivera:** Conceptualization; data curation; formal analysis; funding acquisition; investigation; methodology; project administration; validation; writing–original draft; writing–review and editing. **Supavit Chesdachai:** Formal analysis; investigation; methodology; project administration; validation; writing–original draft; writing–review and editing. **Evan W. Draper:** Conceptualization; data curation; formal analysis; investigation; methodology; validation; writing–original draft; writing–review and editing. **Richard F. Arndt:** Conceptualization; formal analysis; investigation; methodology; validation; writing–original draft; writing–review and editing. **Kristin C. Mara:** Conceptualization; formal analysis; investigation; methodology; visualization; writing–original draft; writing–review and editing. **Maria Gonzalez Suarez:** Conceptualization; investigation; methodology; writing–review and editing. **Raymund R. Razonable:** Conceptualization; data curation; formal analysis; funding acquisition; investigation; methodology; project administration; resources; supervision; writing–original draft; writing–review and editing.

## CONFLICT OF INTEREST STATEMENT

RRR received research funds from Regeneron and Roche on projects not related to this study, as a member of the DSMB for Novartis and is on the Board of Directors of the American Society of Transplantation. CGR is a member of an advisory board for Gilead. All other authors declare no potential conflicts of interest.

### PEER REVIEW

The peer review history for this article is available at https://www.webofscience.com/api/gateway/wos/peer-review/10.1111/irv.13136.

## Supporting information


**Table S1:** Multivariable models among those who survive the initial hospitalization

## Data Availability

The data that support the findings of this study are available from the corresponding author upon reasonable request.
