# Supplementary material for: Clinical outcomes of COVID‐19 treated with remdesivir across the continuum of care
Source: Influenza Other Respir Viruses. 2023 May 17;17(5):e13136. doi: 10.1111/irv.13136 (PMC10205088; doi:10.1111/irv.13136)
Supplement: Supplementary file 1 — Table S1: Multivariable models among those who survive the initial hospitalization [file IRV-17-e13136-s001.docx]

**Supplemental Table 1: Multivariable models among those who survive the initial hospitalization**

|  | Abnormal LFTs | | Subsequent ED/Hospital Encounter | | Death within 28 days  (all-cause) | |
| --- | --- | --- | --- | --- | --- | --- |
|  | Odds Ratio (95% CI) | p-value | Hazard Ratio (95% CI) | p-value | Odds Ratio (95% CI) | p-value |
| **Group** |  |  |  |  |  |  |
| Inpatient/Complete | Reference |  | Reference |  | Reference |  |
| Inpatient/Incomplete | 0.61 (0.46-0.80) | <0.001 | 1.59 (0.93-2.74) | 0.091 | 3.33 (1.91-5.80) | <0.001 |
| Outpatient/Complete | 0.54 (0.43-0.66) | <0.001 | 1.75 (1.15-2.64) | 0.009 | 0.69 (0.28-1.68) | 0.41 |
| Outpatient/Incomplete | 0.46 (0.19-1.14) | 0.095 | 0.67 (0.05-10.05) | 0.77 | 1.66 (0.09-29.98) | 0.73 |
| **Age (per 10 years)** | 0.91 (0.87-0.97) | 0.001 | 1.08 (0.95-1.22) | 0.23 | 2.47 (2.03-3.00) | <0.001 |
| **Sex** |  |  |  |  |  |  |
| Female | Reference |  | Reference |  | Reference |  |
| Male | 1.33 (1.14-1.56) | <0.001 | 1.34 (0.95-1.90) | 0.10 | 0.95 (0.62-1.45) | 0.80 |
| **MASS Score (per 1 point)** | 0.90 (0.87-0.92) | <0.001 | 1.06 (1.00-1.12) | 0.065 | 0.90 (0.84-0.98) | 0.009 |
| **WHO scale** |  |  |  |  |  |  |
| 3 hospitalized no oxygen | Reference |  | Reference |  | Reference |  |
| 4 - mask nasal canula | 1.24 (1.00-1.55) | 0.055 | 1.41 (0.86-2.32) | 0.18 | 2.01 (0.97-4.17) | 0.060 |
| 5 - high flow noninvasive | 2.16 (1.69-2.77) | <0.001 | 1.25 (0.71-2.19) | 0.45 | 3.44 (1.60-7.38) | 0.002 |
| 6 - Invasive | 5.68 (3.46-9.32) | <0.001 | 0.68 (0.21-2.19) | 0.52 | 5.67 (1.92-16.72) | 0.002 |
| 7 - ECMO | 2.31 (0.30-18.12) | 0.42 | 3.51 (0.20-62.11) | 0.39 | -- | -- |
| No data from metrics | 0.63 (0.21-1.89) | 0.40 | 0.87 (0.06-13.51) | 0.92 | 6.10 (0.82-45.53) | 0.078 |
| **c-statistic** | 0.68 | | 0.61 | | 0.82 | |

**Abbreviations:** ED=emergency department, ECMO= extracorporeal membrane oxygenation, LFTs=liver function tests, MASS=Monoclonal Antibody Screening Score, WHO=World Health Organization
